# Comparable Outcomes for Hematologic Malignancies after HLA-Haploidentical Transplantation with Posttransplantation Cyclophosphamide and HLA-Matched Transplantation

**DOI:** 10.1155/2015/431923

**Published:** 2015-12-02

**Authors:** Shannon R. McCurdy, Ephraim J. Fuchs

**Affiliations:** Sidney Kimmel Comprehensive Cancer Center at Johns Hopkins, Baltimore, MD 21287, USA

## Abstract

The implementation of high-dose posttransplantation cyclophosphamide (PTCy) has made HLA-haploidentical (haplo) blood or marrow transplantation (BMT) a cost effective and safe alternative donor transplantation technique, resulting in its increasing utilization over the last decade. We review the available retrospective comparisons of haplo BMT with PTCy and HLA-matched BMT in adults with hematologic malignancies. The examined studies demonstrate no difference between haplo BMT with PTCy and HLA-matched BMT with regard to acute graft-versus-host disease (aGVHD), nonrelapse mortality, and overall survival. Chronic GVHD occurred less frequently after haplo BMT with PTCy compared with HLA-matched BMT utilizing standard GVHD prophylaxis. In addition, patients with a high risk of relapse by the disease risk index had a suggestion of improved progression-free and overall survival after haplo BMT with PTCy when compared with a historical cohort of HLA-matched BMT in one analysis. Furthermore, in Hodgkin lymphoma relapse and progression-free survival were improved in the haplo BMT with PTCy compared with the HLA-matched BMT cohort. These findings support the use of this transplantation platform when HLA-matched related donors (MRDs) are unavailable and suggest that clinical scenarios exist in which haplo BMT may be preferred to HLA-matched BMT, which warrant further investigation.

## 1. Introduction

HLA-haploidentical (haplo) blood or marrow stem cell transplantation (BMT) has historically been limited by unacceptable rates of graft-versus-host disease (GVHD), graft failure, and nonrelapse mortality (NRM). However, modern transplant techniques, specifically the use of high-dose posttransplantation cyclophosphamide (PTCy) on days +3 and +4, have remarkably reduced GVHD and led to the increasing utilization of haplo donors. The feasibility of haplo BMT has dramatically expanded the donor pool, making allogeneic transplantation available for the vast majority of patients. While clinical trials revealed the safety of the haplo approach with a 1-year NRM of 7% after haplo BMT and a 24% NRM after double umbilical cord blood transplantation (dUCB), the 1-year relapse rates of 45% and 31%, respectively [1], led to concern that haplo BMT with PTCy was associated with a high risk of relapse. However, the inflated rate may be more apparent than real, as the observed lower incidence of NRM puts a greater pool of patients at risk of relapse. The ease of application, the reduced cost, and the ready availability of haplo donors have led to the widespread adoption of haplo BMT with PTCy as an alternative donor approach. With its expanded use, an increasing number of retrospective studies (Table 1) have been published showing the safety and efficacy of this transplant platform in adults with hematologic malignancies (two of the examined studies contained a small number of adolescent patients) [2, 3]. We review the available publications that compare haplo BMT with PTCy and HLA-matched BMT in an effort to understand the role of haplo BMT and the prioritization of graft type.

## 2. Graft-versus-Host Disease and Immunosuppression Discontinuation

The majority of the reviewed studies showed that the incidence of acute (a) GVHD was either similar [2, 4, 5] or significantly lower after haplo BMT with PTCy (*p* < 0.001) [3, 6] compared with HLA-matched BMT. The cumulative incidence of grades II–IV aGVHD ranged from 24 to 50% after HLA-matched related donor (MRD), 19% to 50% after HLA-matched unrelated donor (MUD), and 14% to 43% after haplo BMT [2–7]. Grades III-IV aGVHD rates were similarly low after MRD, MUD, and haplo BMT, ranging from 4 to 8%, 4 to 13%, and 0 to 11%, respectively [4–6].

The incidence of chronic (c) GVHD was either significantly lower [5, 7] or tended towards being lower [2–4, 6] after haplo compared with HLA-matched donor BMT. Cumulative incidences of moderate or severe cGVHD were 29%, 22%, and 15% (*p* = 0.053) [3], and extensive cGVHD were 54%, 54%, and 38% (*p* < 0.05) [5] for MRD, MUD, and haplo BMT with PTCy, respectively. When transplants only using BM grafts were compared in one analysis, there was no difference in cGHVD rates after MUD and haplo BMT using either myeloablative (MAC) or reduced intensity conditioning (RIC) [6]. However, in another study, when only transplants using peripheral blood stem cell (PBSC) grafts were compared, the 2-year incidence of moderate-severe cGVHD was 45% after MRD, 48% after MUD, and 25% after haplo (*p* = 0.01 for haplo compared with MRD and *p* = 0.002 for haplo versus MUD) [7]. In keeping with the finding of reduced cGVHD, haplo BMT patients were also more likely to discontinue immunosuppression in both univariable analysis at 1 year (81% compared with 55% in the MRD patients (*p* < 0.001)) [3] and multivariable analysis (*p* = 0.04, *p* < 0.001) [2, 7], in the studies that examined this outcome.

## 3. Immune Reconstitution and Infection

While haplo patients were more likely to have received bone marrow (BM) grafts, which have been associated with engraftment delays [8, 9], neutrophil recovery was similar after haplo BMT with PTCy and HLA-matched BMT. There were low rates of graft failure and time to neutrophil engraftment was similar (18 days in both) [3] or slightly delayed (18 compared with 13 days [4] or 16 compared with 14 [7]) after haplo BMT with PTCy and HLA-matched BMT. In one study, neutrophil recovery was no different after RIC MUD and RIC haplo BMT; however, Day 30 neutrophil recovery was 97% after MAC MUD compared with 90% after MAC haplo BMT, respectively (*p* = 0.02) [6]. Bashey et al. compared neutrophil and platelet engraftment among haplo BMT patients who received either PBSC grafts or BM grafts and found no difference in time to recovery by graft source (16 days to neutrophil engraftment and 26 days to platelet engraftment in both groups) [7]. Immune reconstitution was different at early time points after HLA-matched and haplo BMT, with a decrease in CD3^+^ and natural killer (NK) cell counts at Day 30 [4] and CD4^+^ counts at Day 50 [3] in the haplo cohort. However, there were no differences in CD4^+^, CD3^+^, or NK cell counts after these early time points. CD20^+^ cell counts were similar across transplantation techniques at all time points examined [4].

There was either a trend to an increase [4] or a significant increase [3] in cytomegalovirus (CMV) reactivation after haplo BMT with PTCy compared with MRD and MUD BMT. CMV reactivation rates ranged from 48 to 58%, 54 to 60%, and 71 to 74% after MRD, MUD, and haplo BMT, respectively. Epstein-Barr virus reactivation was either similar with no cases [4] or higher after haplo at 10% compared with 2% after MRD [3]. However, there were no deaths due to posttransplantation lymphoproliferative disease in either cohort of these studies [3, 4].

## 4. Nonrelapse Mortality

Nonrelapse mortality (NRM) was either not significantly different [3–5, 7, 10] or significantly lower (*p* = 0.02) [2] after haplo compared with MRD BMT. NRM at 1 year ranged from 6% to 24% for MRD, 10% to 35% for MUD, and 4% to 24% for haplo BMT with PTCy (Table 2) [4, 5, 10, 11]. Importantly, NRM was comparable across graft types when conditioning intensity was either similar [3, 5, 6] or more intense [4] for patients undergoing haplo allografting with PTCy. In an analysis that included five graft sources, haplo, MRD, and MUD BMT had equivalent NRM; however, dUCB and HLA-mismatched unrelated donor (mmUD) BMT were both associated with higher NRM [3].

## 5. Relapse

When examining outcomes for patients with any hematologic malignancy diagnosis who underwent MRD, MUD, or haplo BMT there was no difference in relapse incidence between the graft types [3, 5, 7]. This was notable given the less frequent use of MAC [5, 7] and PBSC grafts [3, 5, 7] and/or the evidence of more advanced disease [3] in the haplo compared with MRD or MUD BMT cohorts in these studies. Raiola et al. also examined outcomes by disease status and showed a tendency towards less relapse in patients with early phase disease (first or second complete remission) after haplo BMT with PTCy at 18% compared with 36% after MRD BMT (*p* = 0.09), with no difference in relapse incidence for patients beyond second complete remission (*p* = 0.60) [3].

Several studies looked at disease specific outcomes. An analysis of acute myelogenous leukemia (AML) and myelodysplastic syndrome (MDS) patients that utilized similar conditioning platforms across graft types found that the relapse rate was not significantly different after MRD, MUD, or haplo BMT at 28%, 23%, and 33% (*p* = 0.75) [4]. In AML, 3-year relapse risks after MAC MUD and MAC haplo were similar, but rates were lower after NMA MUD compared with NMA haplo BMT. The difference in the NMA cohorts may in part be explained by the longer time from diagnosis to transplantation, poorer performance status scores, and higher proportion of patients transplanted beyond first complete remission (despite no difference in disease risk index between the groups) in the haplo BMT compared with the MUD cohort [6]. Patients with peripheral T-cell lymphoma also had equivalent 1-year cumulative incidence of relapse after MAC MRD at 38% compared with 34% after NMA haplo BMT [10], which is notable given the decreased conditioning intensity in the haplo BMT with PTCy cohort. Notably, in a study of Hodgkin lymphoma, the occurrence of relapse or progressive disease was significantly lower after haplo BMT with PTCy at 40% compared with 56% (*p* = 0.01) and 63% (*p* = 0.03) after MRD and MUD, respectively [2].

## 6. Progression-Free and Overall Survival

Progression-free survival (PFS) and disease-free survival (DFS) were similar after both haplo and HLA-matched BMT in the studies that included either a variety of hematologic malignancies [3, 5, 7, 11] or AML and MDS [4] ranging from 30 to 40% at 3 years [4, 11]. One analysis looked at PFS by disease risk index (DRI) [12] and found that patients with low-risk and intermediate-risk disease had similar PFS after haplo BMT with PTCy and HLA-matched BMT at 65% and 66%, and 39% and 31%, respectively. There was, however, a suggestion of improved outcomes in patients with high or very high-risk disease after haplo BMT with a 3-year PFS of 25% compared with 15% in the HLA-matched setting [11]. In another report, early phase disease was associated with a tendency towards improved DFS at 60% after haplo compared with 38% for MRD, 25% for MUD, 40% for mmUD, and 38% for UCB BMT (*p* = 0.10) [3]. For advanced phase disease, DFS was no different at 32% for haplo, 22% for MRD, 39% for MUD, 18% for mmUD, and 28% for UCB transplantation (*p* = 0.60).

In Hodgkin lymphoma an improvement in PFS was seen after haplo at 51% compared with 23% and 29% after MRD (*p* = 0.0008) and MUD (*p* = 0.03) BMT, respectively [2]. In peripheral T-cell lymphoma, despite the higher median age in the haplo cohort (59 years compared with 46 years), there was no difference in PFS after MRD BMT and haplo BMT with PTCy [10].

Overall survival (OS) was not significantly different in the majority of the analyses and ranged from 53 to 76% after MRD, 58 to 67% after MUD, and 58 to 64% after haplo BMT at 2 years (Table 2) [2, 5]. Three-year OS by DRI was 70% and 73% for low-risk patients, 47% and 49% for intermediate-risk patients, and 25% and 37% for high or very high-risk patients, after HLA-matched and haplo BMT, respectively. In a comparison of haplo, MRD, MUD, mmUD, and UCB transplantation, there was no difference in 4-year actuarial survival at 53%, 45%, 43%, 40%, and 34% (*p* = 0.10), respectively. However, UCB BMT had inferior survival in multivariable analysis (*p* = 0.03), with haplo and MRD having similar survival (*p* = 0.80) [3]. Finally, 4-year OS in advanced disease by BMT platform was 47%, 30%, 31%, 20%, and 27%, after haplo, MRD, MUD, mmUD, and UCB transplantation, respectively (*p* = 0.20) [3].

## 7. Discussion

PTCy has decreased the incidence of GVHD, graft failure, and NRM associated with haplo BMT and led to its increasing adoption for patients without an HLA-matched donor. We review the existing retrospective comparisons of HLA-matched BMT and haplo BMT with PTCy in adults with hematologic malignancies. With the use of PTCy based GVHD prophylaxis, rates of aGVHD after haplo BMT appear comparable to that after MRD BMT utilizing standard prophylaxis. While we found similar rates of aGVHD, cGVHD incidence was reduced in the haplo compared with the MRD BMT cohorts. We believe this finding is attributable to PTCy, the use of which was limited to the haplo cohorts in these studies. PTCy, when given early posttransplant, is cytotoxic to alloreactive T-cells that would eventually contribute to cGVHD development. Traditional immunosuppressants, such as calcineurin inhibitors, methotrexate, or mycophenolate mofetil, only inhibit the immune system and flare of GVHD can occur with their cessation. With PTCy, cGVHD prevention is mediated early after transplant and does not require continued use of immunosuppression. Engraftment and immune reconstitution of CD3^+^, CD4^+^, and NK cells also appear similar in haplo and MRD BMT after the early posttransplant time period. While the slight delay in neutrophil engraftment and reduction in T-cell counts before Day 50 may be associated with either the haplo graft or the PTCy, it is possible that the use of BM as a stem cell source, which has been associated with engraftment delay [8, 9] and was used preferentially in the haplo cohort, may have contributed. However, the one study that compared neutrophil engraftment after haplo PBSC and haplo BM allografting found no difference in time to neutrophil or platelet recovery [7].

With comparable aGVHD and graft failure rates and a reduced incidence of cGVHD, we would expect a similar NRM. As demonstrated in the early studies of haplo BMT with PTCy, NRM rates were low in these reports, comparable to that seen after MRD BMT. As such, there is now strong evidence for the safety of this transplant platform.

Relapse rates, on the other hand, were a purported weakness associated with haplo BMT with PTCy, owing to the original Phase II study, which found a 45% relapse rate at 1 year [1]. This was an unexpected finding given that the increasing HLA-mismatch could potentially lead to more graft-versus-tumor effects and less relapse after haplo BMT compared with HLA-matched grafts. Critics believed that the PTCy inhibited not only the negative effects of HLA-mismatch, namely, GVHD and graft failure, but the positive graft-versus-tumor effects as well. After reviewing the existing literature comparing HLA-matched and haplo BMT there appears to be no difference in relapse rate in the majority of these retrospective studies. In fact, in certain diseases, relapse may be decreased after haplo BMT. This has been suggested in a study of Hodgkin lymphoma in which relapse and PFS were significantly improved in the haplo cohort compared with the MRD and MUD cohorts, despite the use of BM as a graft source in the haplo cohort and PBSC in the HLA-matched cohort (PBSC have been associated with reduction in relapse in prior analyses [13]). In a single armed study of haplo BMT with PTCy for relapsed Hodgkin lymphoma after prior autologous grafting, Raiola et al. reported a 3-year EFS of 63% [14]. These results support the efficacy of haplo BMT for patients with poor risk Hodgkin lymphoma. Furthermore, MAC has been associated with decreased relapse and increased NRM, equating to no difference in OS when compared with NMA conditioning in prior studies [15]. Despite decreasing conditioning intensity, relapse in peripheral T-cell lymphoma patients was equivocal after with MAC MRD and NMA haplo, suggesting that haplo BMT with PTCy may play an important role in relapse reduction in this disease. However, given the limited and retrospective nature of this data, further study is warranted to better clarify the hierarchy of haplo in transplantation for lymphoma and the effects of haplo BMT with PTCy on relapse.

Outside of lymphoma, stage or risk of disease may also present a scenario in which haplo grafts may be preferred. In the analysis of outcomes by the DRI, there was a suggestion of improved PFS and OS in high and very high-risk disease (risk is determined by disease characteristics and disease stage at transplantation) [11, 12]. This finding reflects the very early clinical data of haplo BMT before the era of PTCy, in which patients with early phase disease did worse after haplo compared with MRD BMT, owing to an increased NRM. However, survival in patients with advanced leukemia after haplo BMT was more similar to those after MRD BMT in that study [16]. The difference in outcomes by disease risk may have been due to a higher risk of death from relapse in high-risk patients. Therefore, the outcomes of high-risk patients depended less on the risk of NRM and more on relapse reduction, which was more effective after haplo BMT. However, Raiola et al. found a trend towards reduction of relapse in early phase disease after haplo BMT with PTCy compared with MRD BMT and similar relapse rates in advanced disease [3]. In the early phases of MAC haplo BMT with PTCy, patients with active leukemia were transplanted and outcomes were poor due to progressive disease early after BMT. As a result, Johns Hopkins adopted a policy to avoid transplantation of patients not in remission. Similarly, with HLA-matched transplant platforms, active disease at the time of transplantation has been associated with poor outcomes, especially in the setting of RIC [17]. Given these contradictory findings, the preferential use of haplo grafts for a given disease stage or risk warrants further investigation.

In all, OS and PFS were not different after haplo and MRD BMT in the studies that compared the two transplant platforms. This suggests that, at a minimum, haplo is an acceptable alternative to HLA-matched transplantation, but further studies are needed to elucidate the clinical scenarios in which haplo BMT with PTCy may be preferred. In the future, other donor factors such as age, sex mismatch, ABO match, CMV compatibility, or NK cell alloreactivity may be more critical than HLA match for donor selection.

## Figures and Tables

**Table 1:** Summary of included studies.

Study	Disease	HLA type and patient number	Conditioning regimens	GVHD prophylaxis	Graft source
McCurdy et al. Blood 2015 [11]	Hematologic malignancies	Haplo *n* = 372	Haplo: Flu 30 mg/m^2^ D-6–2 Cy 14.5 mg/kg D-6-5 TBI 200 cGY D-1	Haplo: PTCy 50 mg/kg D3 and D4, MMF D5–35, Tac D5–90 or 180	BM
		Historical MRD or MUD cohort *n* = 614^*∗*^	MRD/MUD: Flu 120 mg/m^2^ Bu 3.2–6.4 IV mg/kg ±ATG	MRD/MUD: CNI + MTX ± sirolimus or CSP with MMF	BM or PBSC

Ciurea et al. Blood 2015 [6]	AML	MUD *n* = 1982	MAC: TBI/Cy	MUD: CNI + MTX or MMF	PBSC > BM
		Haplo *n* = 192	TBI/Flu Bu/Cy Mel/Thiotepa/Flu Bu/Flu Bu/Flu/ATG Bu/Thiotepa/Flu NMA: Flu/Cy/TBI	Haplo: PTCy 50 mg/kg D3 and D4, MMF, and CNI	BM > PBSC

Bashey et al. BBMT [7]	Hematologic malignancies	MRD *n* = 181 MUD *n* = 178	MRD or MUD: Bu/Cy	MRD or MUD: Tac + MTX ± ATG ± alemtuzumab	PBSC ≫ BM
		Haplo *n* = 116	Flu/Bu		
			Flu/Bu/Cy Flu/Mel Etop/TBI		
			Flu/Cy Flu/Cy/TBI Cy/TBI Mel/TBI Flu/TBI Bu/Cy/Etop NMA haplo: Flu/Cy/TBI MAC haplo: Flu/Bu/Cy Flu/TBI	Haplo: PTCy 50 mg/kg D3 and D4, MMF, and CNI	NMA haplo: BM ≫ PBSC MAC haplo: PBSC only

Raiola et al. BBMT 2014 [3]	Hematologic malignancies	MRD *n* = 176 MUD *n* = 43 mmUD *n* = 43 UCB *n* = 105 Haplo *n* = 92	MAC: Thio/Bu/Flu Bu/Cy Flu/TBI Cy/TBI RIC: Flu/Cy/TBI Thio/Cy ± Mel	MRD: CSP + mini-MTX MUD: CSP + mini-MTX + ATG UCB: CSP + MMF + ATG Haplo: PTCy 50 mg/kg D3 and D4 + CSP + MMF	BM PBSC UCB

Bashey et al. JCO 2013 [5]	Hematologic malignancies	MRD *n* = 117 MUD *n* = 101	RIC or MAC	Standard regimens	PBSC or BM
		Haplo *n* = 53	NMA haplo: Flu 30 mg/m^2^ D-6–2 Cy 14.5 mg/kg D-6-5 TBI 200 cGY D-1 or	Haplo: PTCy 50 mg/kg D3 and D4, MMF D5–35, and Tac D5–180	BM for NMA
			MAC haplo: Flu 25 mg/m^2^ D-6–2, Bu 110–130 mg/m^2^ IV D-7–4, Cy 14.5 mg/kg D-3-2		PBSC for MAC

Di Stasi et al. BBMT 2014 [4]	AML or MDS	MRD *n* = 87 MUD *n* = 108	MRD/MUD: Flu 120–160 mg/m^2^ in 4 daily doses Mel 140 mg/m^2^ or 100 mg/m^2^	MRD: Tac + mini-MTX MUD: Tac + mini-MTX + ATG	BM or PBSC
		Haplo *n* = 32	Haplo: above + thiotepa 5–10 mg/kg	Haplo: PTCy 50 mg/kg D3 and D4, MMF D5–100, Tac D5–180	BM or PBSC

Kanakry et al. BBMT 2013 [10]	Peripheral T-cell lymphoma	MRD *n* = 22	Bu/Cy	MRD: CSP or PTCy 50 mg/kg D3 and D4 ±MMF ±Tac	
		Haplo *n* = 22	Cy/TBI Flu/TBI Bu/Flu Flu/Cy/TBI Fly/Cy/TBI Bu/Cy Cy/TBI	Haplo: PTCy 50 mg/kg D3 and D4, MMF, Tac, or CSP	BM (1 PBSC)

Burroughs et al. BBMT 2008 [2]	Hodgkin lymphoma	MRD *n* = 38 MUD *n* = 24	MRD/MUD: TBI 2 Gy TBI 2 Gy + Flu 30 mg/m^2^ days 4–2	MRD/MUD: MMF or CNI	PBSC
		Haplo *n* = 28	Haplo: Flu 30 mg/m^2^ D-6–2 Cy 14.5 mg/kg D-6-5 TBI 200 cGY D-1	Haplo: PTCy 50 mg/kg D3 (±D4), MMF D4 or 5–35, Tac D4, or 5–180	BM

HLA: human leukocyte antigen; GVHD: graft-versus-host disease; haplo: HLA-haploidentical; *n*: number; MRD: HLA-matched related donor; MUD: HLA-matched unrelated donor; Flu: fludarabine; D: day; Cy: cyclophosphamide; TBI: total body irradiation; Bu: busulfan; ATG: antithymocyte globulin; PTCy: posttransplantation cyclophosphamide; MMF: mycophenolate mofetil; Tac: tacrolimus; CNI: calcineurin inhibitor; MTX: methotrexate; CSP: cyclosporine; BM: bone marrow; PBSC: peripheral blood stem cells; AML: acute myelogenous leukemia; MAC: myeloablative conditioning; Mel: melphalan; BBMT: biology of blood and marrow transplantation; Etop: etoposide; NMA: nonmyeloablative; mmUD: HLA-mismatched unrelated donor; UCB: umbilical cord blood; JCO: Journal of Clinical Oncology; MDS: myelodysplastic syndrome.

^*∗*^Armand et al. [12], a disease risk index for patients undergoing allogeneic stem cell transplantation, July 2012; Blood: 120 (4) 905–913.

**Table 2:** Survival outcomes.

Study	NRM		Relapse		PFS or DFS		OS	
	Haplo	Matched (MRD/MUD)	Haplo	Matched (MRD/MUD)	Haplo	Matched (MRD/MUD)	Haplo	Matched (MRD/MUD)
McCurdy et al. Blood 2015 [11]	1 yr: 11%		3 yr: 46%		3 yr: 40%		3 yr: 50%	
			Low: 20%		Low: 65%	66%^*∗*^	Low: 73%	70%
			Int: 48%		Int: 39%	31%	Int: 49%	47%
			High: 67%		High: 25%	15%	High: 37%	25%

Ciurea et al. Blood 2015 [6]	MAC:		MAC:				MAC:	
	1 yr: 12%	14%	3 yr: 44%	39%			3 yr: 45%	50%
	RIC:		RIC:				RIC:	
	1 yr: 6%	16%	3 yr: 58%	42%			3 yr: 46%	44%

Bashey et al. BBMT 2015 [7]	2 yr: 17%	(14%/16%)	2 yr: 29%	(30%/34%)	2 yr: 54%	(56%/50%)	2 yr: 57%	(72%/59%)

Raiola et al. BBMT 2014 [3]	D1000: 18%	(24%/33%)	35%	(40%/23%)	4 yr: 43%	(32%/36%)	4 yr: 52%	(45%/43%)

Bashey et al. JCO 2013 [5]	1 yr: 4%	(10%/10%)	2 yr: 33%	(34%/34%)	2 yr: 60%	(53%/52%)	2 yr: 64%	(76%/67%)

Di Stasi et al. BBMT 2014 [4]	1 yr: 24%	(20%/35%)	1 yr: 33%	(28%/23%)	3 yr: 30%	(36%/27%)		

Kanakry et al. BBMT 2013 [10]	1 yr: 8%	MRD 6%	1 yr: 34%	MRD 38%				

Burroughs et al. BBMT 2008 [2]	2 yr: 9%	(21%/8%)	2 yr: 40%	(56%/63%)	2 yr: 51%	(23%/29%)	2 yr: 58%	(53%/58%)

NRM: nonrelapse mortality; haplo: human leukocyte antigen- (HLA-) haploidentical; matched: HLA-matched; MRD: HLA-matched related donor; MUD: HLA-matched unrelated donor; PFS: progression-free survival; DFS: disease-free survival; OS: overall survival; yr: year; low: low risk by disease risk index; Int: intermediate risk by disease risk index; high: high or very high risk by disease risk index; BBMT: biology of blood and marrow transplantation; D: day; JCO: Journal of Clinical Oncology.

^*∗*^Data based on 614 recipients of reduced intensity conditioning and HLA-matched stem cell transplantation from the original disease risk index study cohort [12] whose outcomes were tabulated and received from P. Armand Dana-Farber Cancer Institute, email, July 24, 2014, personal communication.
